# Calcaneus secundarius – a relevant differential diagnosis in ankle pain: a case report and review of the literature

**DOI:** 10.1186/s13256-015-0595-7

**Published:** 2015-06-02

**Authors:** Daniel Krapf, Sebastian Krapf, Christian Wyss

**Affiliations:** 1 Kantonsspital Aarau, Fusszentrum, Tellstrastrasse, CH-5001 Aarau, Switzerland; 2 Universitäts-Kinderspital beider Basel, Spitalstrasse 33, CH-4056 Basel, Switzerland

**Keywords:** Ankle sprain, Accessory ossicle, Calcaneus secundarius, Anterior process of the calcaneus, Calcaneonavicular coalition

## Abstract

**Introduction:**

Accessory ossicles of the foot are a common finding. Although mostly asymptomatic, they can gain clinical relevance by trauma or stress on the complex biomechanical system of the foot. There are few reports on the entity of symptomatic calcaneus secundarius. Furthermore, the current literature does not address the need for awareness of calcaneus secundarius as a differential diagnosis in cases of persistent posttraumatic ankle pain.

**Case presentation:**

We present the case of a 51-year-old Indo-European man with a medical history of persistent load-dependent ankle pain over 3 decades. At presentation after an acute ankle sprain, we diagnosed a traumatized calcaneus secundarius. Surgical excision led to a complete recovery. More than 1 year postoperative he is still asymptomatic.

**Conclusions:**

With the presented case and review of the literature we demonstrate the clinical relevance of calcaneus secundarius. Depending on size and alignment, calcaneus secundarius can alter the biomechanics in the subtalar region generating pain at the ankle. If a patient has persistent sinus tarsi syndrome, a painful limited subtalar range of motion or repetitive ankle sprains, then calcaneus secundarius should be considered in differential diagnosis. Likewise when a fracture of the anterior process of the calcaneus or a calcaneonavicular coalition is suspected, calcaneus secundarius should be considered a possible diagnosis by all clinicians confronted with foot and ankle pain.

## Introduction

**Figure 1:**
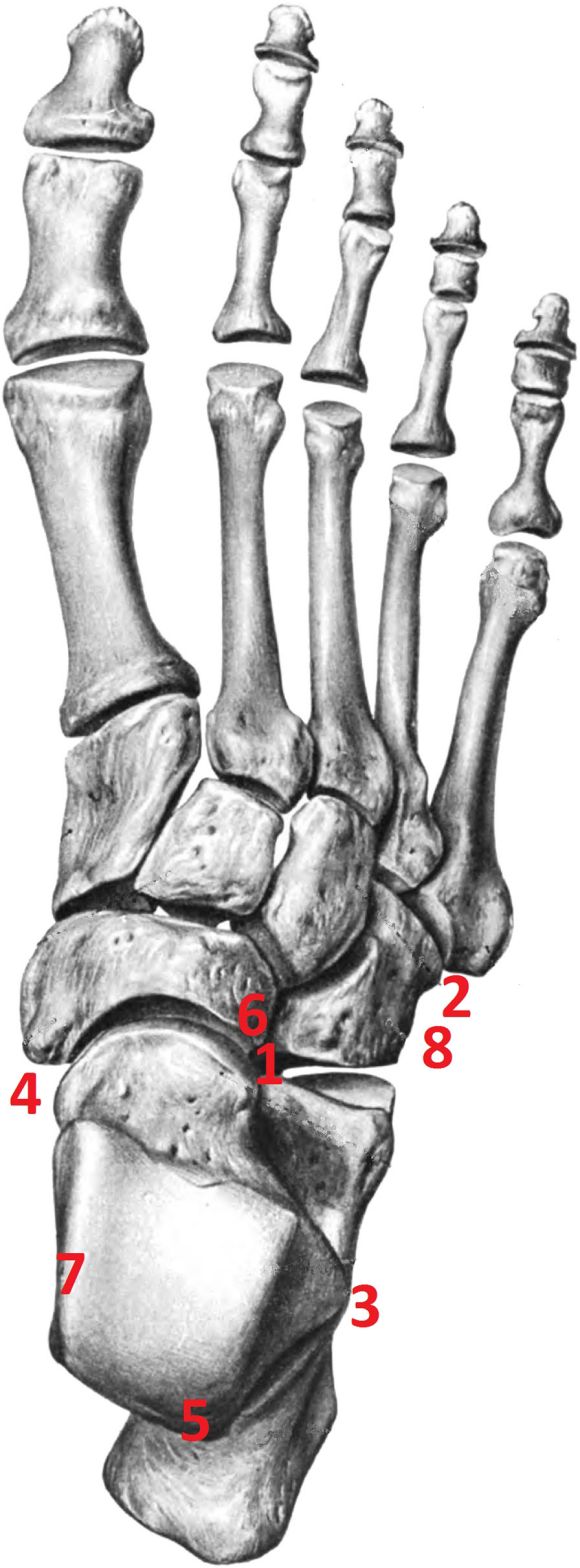
Location of mentioned accessory ossicles of the foot. 1. Calcaneus secundarius, 2. os vesalianum, 3. calcaneus accessorius, 4. os tibiale externum, 5. os trigonum, 6. os cuboideum secundarium (plantar), 7. os sustentaculum secundarium (medial to calcaneus), and 8. os peroneum.

Since the first description of an accessory ossicle of the foot by Vesalius in 1543 [1], accessory bones of the foot have been well documented in the literature, the most common ones being the os tibiale externum, the os trigonum and the os peroneum [2-7] See Figure 1 for anatomic location. The os calcaneus secundarius (CS) is located between the calcaneus, the cuboid, the talus and the navicular bone. Moreover it may form a set of articulations with the cuboid and the talus [8]. CS was first described by Stieda in 1869 [9,10]. The oldest known case is documented in an Egyptian mummy [11,12]. However, reports in the literature are rather sparse [13-23]. The term os CS, sometimes also referred to as os calcis secundarius, was first adopted by Dwight and Piersol in 1907 [24].

The prevalence of CS as described in the literature is uncertain, since it differs significantly depending on the type of study (radiologic or anatomic) [4,25] and on the population studied [25-27]. With a prevalence ranging from 0.14% up to 7% the CS is one of the more infrequently found accessory bones of the foot, which though should not be underestimated [2,4,5,7,12,15,16,26,28-33]. The frequency of CS may vary between different peoples. Silva, for example, reported on a Neolithic-Chalcolithic population in Almada, Portugal, in which an increased incidence of CS was found, namely 8.6% in left and 15.2% in right feet [27]. Although until now no heredity transmission has been proven, these findings raised a discussion about aspects such as geographic isolation and intermarriage in relation to the incidence of CS [26]. Modern industrial populations show a significantly lower incidence of CS (<2%) than nonindustrial groups (>4%) [25]. Laidlaw stated that the low incidence of CS found in anatomic specimens may be due to maceration before examination [18]. Environmental factors and association with type of facet configuration and CS are discussed [25,27]. CS is most often associated with a type I and type II configuration of the middle and anterior facet [7,25,32]. Equally little is known on the developmental aspect of this entity.

Various explanations for the origin of the CS are reported in the literature. One theory explains the development from a secondary center of ossification at the anterior facet of the calcaneus [25]. Gruber stated that a collapse of the tarsal bones is the reason for the accessory tarsal bones [15]. By contrast, Cihak describes the development of the calcaneus in the 5th to 6th embryonic week out of two different parts: a distal fibular and a proximal pisiforme part [34]. In Steiner’s concept of independently developing lateral ray the CS represents a persisting additional third ray [35]. De Cuveland sees CS as a persisting inconstant apophysis of an inconstant processus anterior calcanei [36], while Niederecker assumes a calcification of the calcaneo-navicula part of the ligamentum bifurcatum as the cause of CS [37]. Several authors describe the CS as different developmental stages of a calcaneonavicular coalition [16,36,38]. Another possibility might be trauma to the immature cartilaginous calcaneus as cause of an accessory bone island [25].

The size of CS may vary as well. The average size of this accessory bone is reported to be 3 to 4mm in diameter [4]. The smallest symptomatic CS as reported in the literature are presented in Table 1 and selection of asymptomatic CS are presented in Table 2. Larger examples with maximum 20mm length, 12mm width, and 8mm height, or a pyramidal shape, with 20mm and 13mm being the two maximal side lengths, were described [13,20]. Clinical signs can typically be local pain on weight bearing or on palpation or restricted subtalar motion with or without previous trauma. Persistent pain after an ankle sprain is one of the first mentioned symptoms [17]. In the following we report the case of a patient who was treated unsuccessfully for persistent posttraumatic ankle pain for many years until tomography (CT) at our institution revealed the largest CS ever described. We discuss the possible treatment options for symptomatic CS and compare our method of treatment with published cases. For literature research we used PubMed and Google Scholar (all until November 2014) with the following search terms: “calcaneus secundarius” OR “calcaneus accessorius”. We included all articles providing relevant information for the topic. We checked all reference lists of the included articles for additional relevant studies. Articles published in languages other than English and German were not excluded.

**Table 1:** **Clinically symptomatic cases of calcaneus secundarius as described in the literature**

**Author(s), year of publication and Reference number**	**Size (length/width/height)**	**Identification**
	**[mm]**	
Slomann, 1921 [22]	3/5	Radiological
Krida, 1923 [17]	“Small piece of cancellous bone”	Operative
Naumann, 1955 [21]	“Large”	Radiological
Marti, 1955 [19]	“Round, well-structured piece of bone”	Radiological
Heikel, 1962 [16]	“Piece of bone”	Radiological
Stauss *et al*., 2003 [23]	“Small ossicle distal processus anterior”	Radiological
Ceroni *et al*., 2006 [13]	20/12/8	Operative
Ersen, 2013 [14]	“Ovoid cortical bone fragment with blunt edges”	Radiological
Baghla *et al*., 2010 [42]	15/12/10	Radiological
This work	22/18/16 (magnetic resonance imaging), 20/14/14 (computed tomography)	Radiological

**Table 2:** **Selection of clinically asymptomatic cases of calcaneus secundarius as described in the literature**

**Author(s), year of publication and Reference number**	**Size (length/width/height)**	**Identification**
	**[mm]**	
Stieda, 1869 [9]	3 cases, maximum size 15/8/5	Anatomical
Gruber, 1871 [15]	12/9/7	Anatomical
Pfitzner and Schwalbe, 1892 [6]	5 cases, maximum size 15/8/5	Anatomical
Laidlaw, 1905 [18]	3 cases, maximum 16.5/6.5/6.0	Anatomical
Mercer, 1931 [20]	4 cases, two maximum side lengths of a pyramid 20/13	Anatomical

## Case presentation

**Figure 2:**
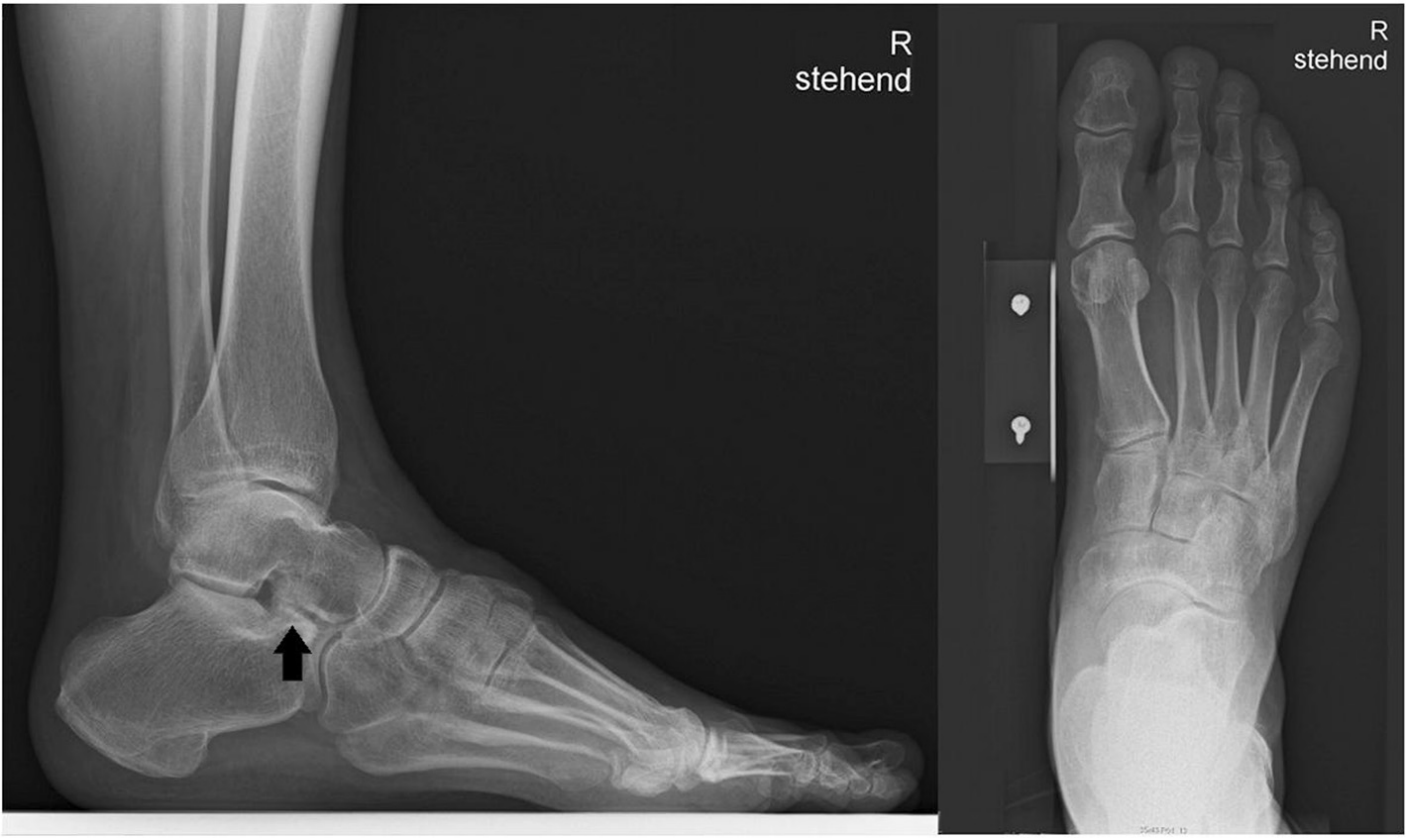
Lateral and dorsoplantar X-ray of the involved foot preoperatively. The arrow is pointing to the calcaneus secundarius.

**Figure 3:**
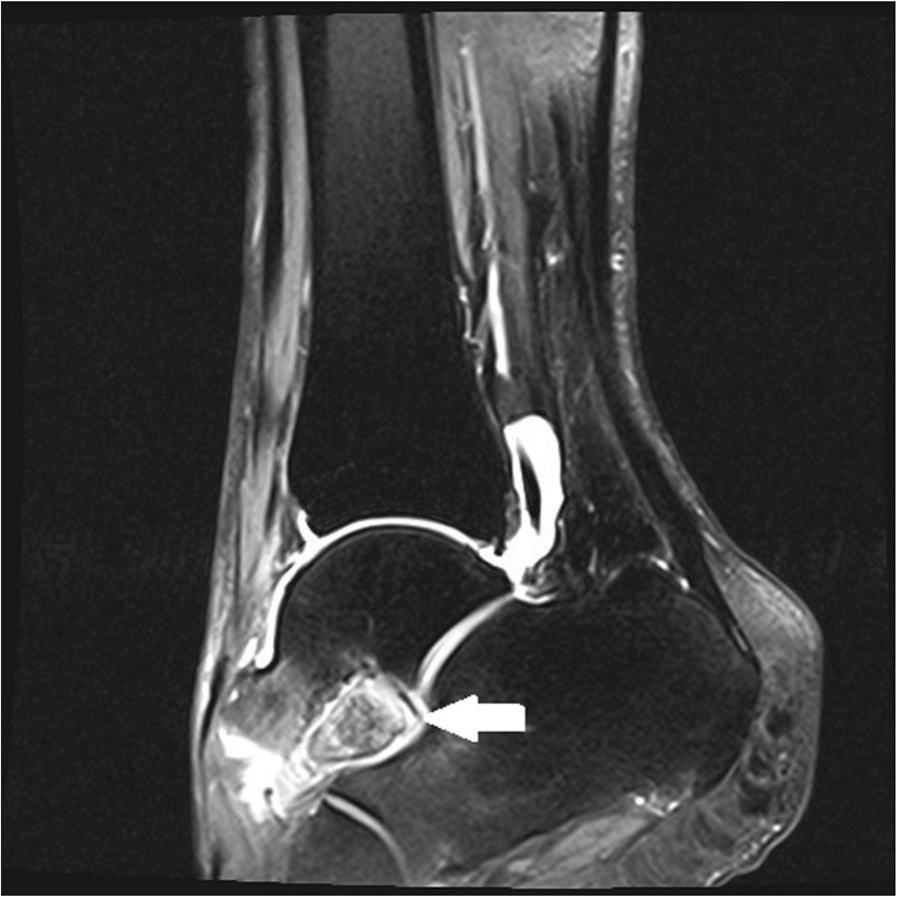
Sagittal magnetic resonance imaging of the foot, T2-weighted image showing the calcaneus secundarius (arrow) preoperatively.

**Figure 4:**
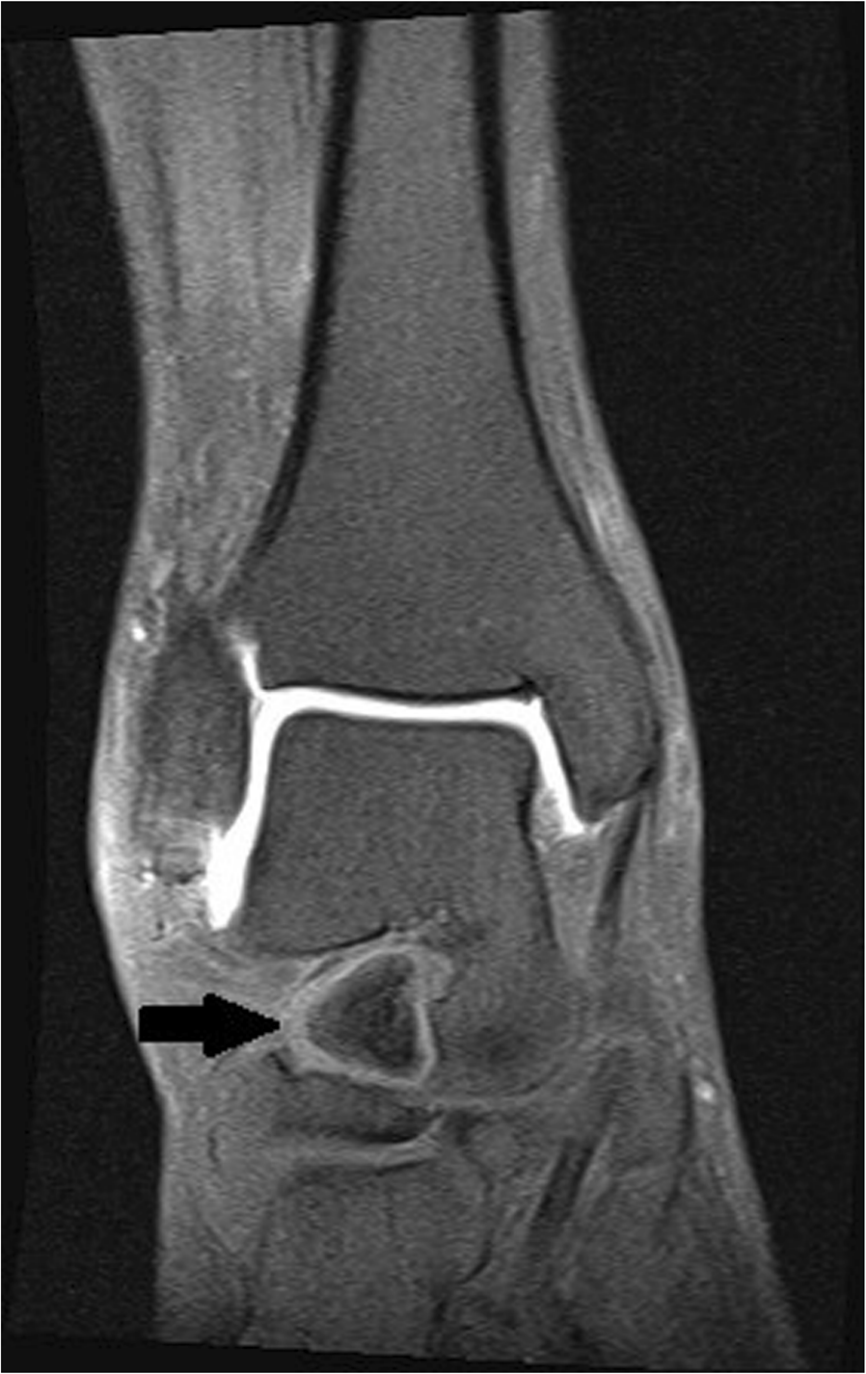
Frontal magnetic resonance imaging of the foot, T1-weighted image showing the calcaneus secundarius (arrow) preoperatively.

**Figure 5:**
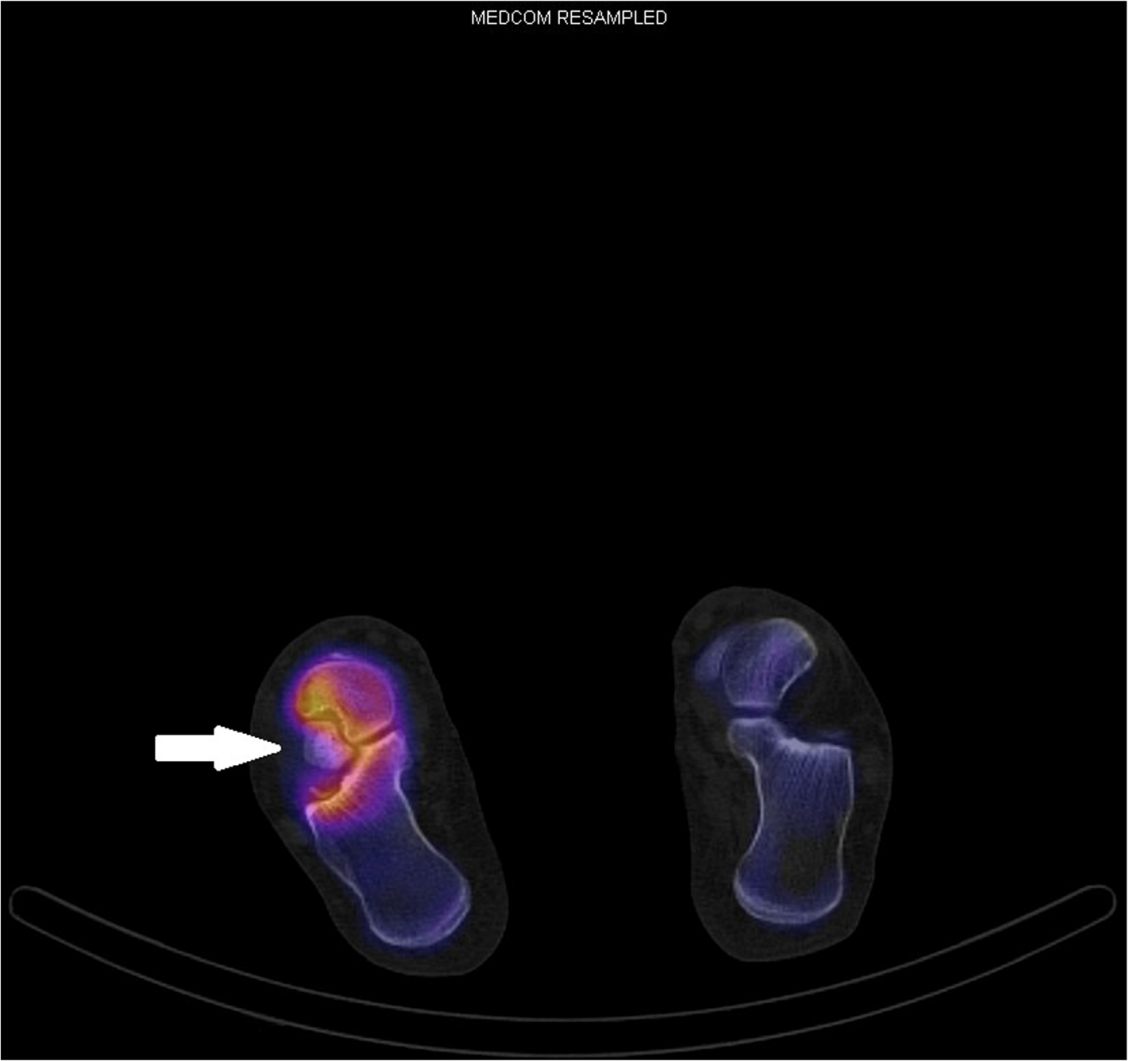
Preoperative single-photon emission computed tomography-computed tomography scan, transversal plane. Activity around the calcaneus secundarius (arrow) visible.

**Figure 6:**
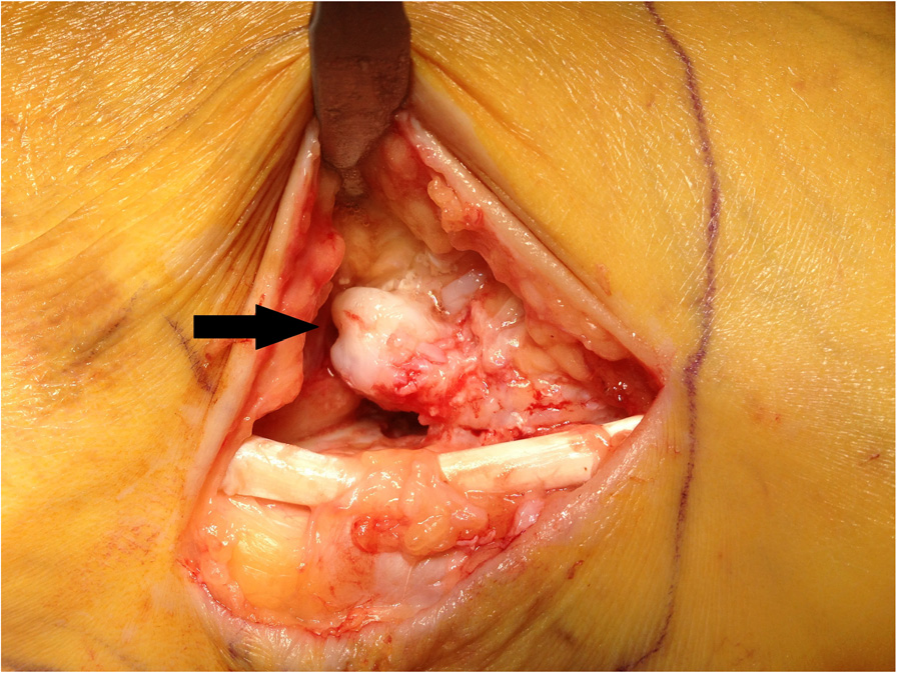
Intraoperative finding of the calcaneus secundarius (arrow).

**Figure 7:**
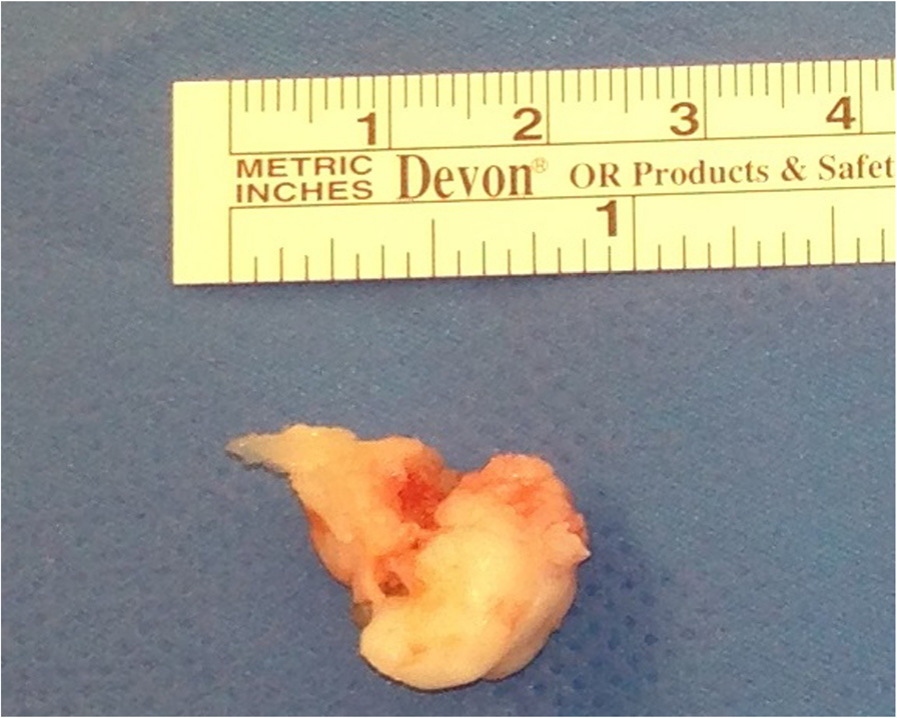
Excised main fragment of the calcaneus secundarius.

We report the case of a symptomatic os CS with a maximum length of 22mm, width 18mm, and height 16mm in magnetic resonance imaging (MRI; see Figures 2,3,4,5,6 and 7) in a 51-year-old Indo-European man. He presented to our out-patient clinic with 20 months’ history of persistent, disabling pain after a right-sided ankle sprain. From his past medical history he reported an ankle sprain of the same side some 30 years previously with persisting load-dependent pain ever since. These symptoms precluded him from further participation in regular sport activity such as soccer or track and field training. He now was diagnosed for a non-displaced fracture of the talar neck (MRI) and treated conservatively without weight bearing and immobilization in a walker boot for 12 weeks. Still with progressive weight bearing in the course of treatment he noted a recurrence of mid- and rear foot pain in the subtalar region. Several consecutive infiltrations of his ankle and subtalar joint were performed without long-term improvement. In MRI a mass situated in the sinus tarsi was interpreted as a calcified hematoma or scar tissue (see Figures 3 and 4). Consecutively, sinus tarsi syndrome was diagnosed.

When he presented to our center for foot and ankle surgery, he had painful, restricted subtalar motion and local sensitivity on palpation at the distal sinus tarsi region. We reviewed the latest MRI and stated that he had a traumatized os CS, which was larger than all cases reported so far. We confirmed the diagnosis by single-photon emission computed tomography-CT imaging and local infiltration. This time infiltration with a combination of steroid and local anesthetic kept him pain-free for 1 month, with complete return of symptoms within several weeks.

Surgical excision through a lateral approach was therefore performed. Approximately 1 year after excision he ambulates without pain (American Orthopaedic Foot and Ankle Society) hindfoot score 95/100, preoperative 24/100) [39] and is able to work full-time as a construction worker in loaded positions in adapted solid foot gear. He also started recreational sport activities again.

## Discussion

To date there are only a few cases of symptomatic os CS published in the literature [13,14,16,17,19,21,23], of which none are as large as the one we describe (see Table 1). However, all these cases outline the importance of the CS as a potential differential diagnosis in athletic foot injuries or persistent ankle pain [17,32,40,41]. CS is not to be confused with the more often reported entity of symptomatic calcaneus accessorius [4,42-47]. Other entities to be considered are the os cuboideum secundarium [4,30] and the os sustentaculum secundarium [2,4,36]. In a posttraumatic setting CS can be mistaken for an anterior process fracture of the calcaneus [17,32,40,41]. Therefore distinct nomenclature has to be defined [48]. Calcaneonavicular coalition is another radiological and clinical differential diagnosis [16,36,38,49].

Although some cases have been reported in the literature, there is no consensus on therapy or conclusive reports on the result of different therapeutic options.

Radiologic diagnosis is best performed using lateral oblique views of the foot. After trauma CS has to be considered; we recommend plain radiographs including lateral oblique views to the standard weight-bearing lateral and dorsoplantar views. If this set of images fails to be significant, tomography (MRI or CT) should be performed to confirm or exclude the diagnosis. For difficult cases and questionable calcaneonavicular coalition or fracture, the literature advises use of MRI, CT or scintigraphy [5,41,49].

Considering the appropriate therapy, Ceroni *et al*. and Krida chose an operative approach similar to ours with good clinical results [13,17]. Heikel’s patient had improvement of symptoms after excision with persistence of pain after heavy work [16]. The patient of Ersen *et al*. recovered completely under symptomatic therapy (non-steroidal anti-inflammatory drugs and mobilization after 1 month) [14].

## Conclusions

CS is a relevant differential diagnosis in persistent pain after a supination trauma to the ankle. Traumatized CS should be considered in supposed case of fracture of the anterior process of the calcaneus, calcaneonavicular coalition, sinus tarsi syndrome and persistent pain after an ankle sprain.

Given the small number of patients reported with symptomatic CS, guidelines considering diagnostics and therapy are lacking. Therefore awareness is specifically important for all professionals treating patients for ankle sprains. Since the benefit of surgical treatment so far cannot be ascertained, we recommend conservative treatment as the first step of therapy, combining restriction of weight bearing and immobilization for several weeks in addition to symptomatic therapy. In case of failure, local steroid infiltration can be considered. We do advise excision after several months of unsuccessful conservative treatment. Depending on degenerative involvement of the subtalar joint, it has to be decided if subtalar fusion is necessary simultaneously.

## Consent

Written informed consent was obtained from the patient for publication of this case report and any accompanying images. A copy of the written consent is available for review by the Editor-in-Chief of this journal.

## Abbreviations

CS: Calcaneus secundarius
CT: Computed tomography
MRI: Magnetic resonance imaging
